# Individual 3D-printed fixation masks for radiotherapy: first clinical experiences

**DOI:** 10.1007/s11548-021-02393-2

**Published:** 2021-05-22

**Authors:** M. Mattke, D. Rath, M. F. Häfner, R. Unterhinninghofen, F. Sterzing, J. Debus, F. L. Giesel

**Affiliations:** 1 Department of Radiation Oncology, Heidelberg University Hospital, Heidelberg, Germany; 2 Heidelberg Institute of Radiation Oncology (HIRO), Heidelberg University Hospital, Heidelberg, Germany; 3 National Center for Tumor Diseases (NCT), Heidelberg University Hospital, Heidelberg, Germany; 4 Clinical Cooperation Unit Radiation Oncology, German Cancer Research Center (DKFZ), Heidelberg, Germany; 5 Department of Radiation Oncology, Heidelberg Ion-Beam Therapy Center (HIT), Heidelberg University Hospital, Heidelberg, Germany; 6 Department of Nuclear Medicine, Heidelberg University Hospital, Heidelberg, Germany; 7 Radioonkologie Speyer, Speyer, Germany; 8 Radioonkologie Kempten, Kempten, Germany; 9 Institut Für Anthropomatik Und Robotik, Karlsruher Institut Für Technologie (KIT), Karlsruhe, Germany; 10 Institut Für Robotik Und Mechatronik, Deutsches Zentrum Für Luft- Und Raumfahrt, Oberpfaffenhofen-Weßling, Germany

**Keywords:** Immobilization, Radiotherapy, 3D-printing, Setup accuracy, Head mask

## Abstract

**Purpose:**

To show the feasibility of 3D-printed fixation masks for whole brain radiation therapy in a clinical setting and perform a first comparison to an established thermoplastic mask system.

**Methods:**

Six patients were irradiated with whole brain radiotherapy using individually 3D-printed masks. Daily image guidance and position correction were performed prior to each irradiation fraction. The vectors of the daily position correction were compared to two collectives of patients, who were irradiated using the standard thermoplastic mask system (one cohort with head masks; one cohort with head and neck masks).

**Results:**

The mean systematic errors in the experimental cohort ranged between 0.59 and 2.10 mm which is in a comparable range to the control groups (0.18 mm–0.68 mm and 0.34 mm–2.96 mm, respectively). The 3D-printed masks seem to be an alternative to the established thermoplastic mask systems. Nevertheless, further investigation will need to be performed.

**Conclusion:**

The prevailing study showed a reliable and reproducible interfractional positioning accuracy using individually 3D-printed masks for whole brain irradiation in a clinical routine. Further investigations, especially concerning smaller target volumes or other areas of the body, need to be performed before using the system on a larger basis.

## Introduction

Accurate patient positioning and fixation are crucial in conformal radiotherapy to guarantee the best possible dose coverage in the target region while sparing healthy tissue. In particular, this applies to tumors in the head and neck area where many radiosensitive organs-at-risk (OAR) are found in close proximity, e.g., the brain stem, the optic system, or the pituitary gland [3, 11, 21]. In an era of high conformal radiotherapy with steep dose gradients a higher sensitivity to setup uncertainties arises. Therefore, accurate patient positioning is more important than ever before [9, 10].

Currently, most patients undergoing radiotherapy of the brain or head and neck region are immobilized using thermoplastic mask systems [17]. Other devices like scotch cast masks are rarely used in current times. The most important feature of all those devices is to create a reliable inter- and intrafractional positioning accuracy, which has been evaluated before with inaccuracies < 5 mm [2, 6, 8, 20]. Though the most commonly used thermoplastic mask systems are a reliable and safe immobilization device, they are prone to trigger stress or fear. According to Nixon et al., approximately 26% of patients suffer from anxiety concerning the mask system [15, 16, 19]. In our experience, even non-claustrophobic patients experience problems as they often report that the masks are uncomfortable. Especially pressure or even pain in the area of the nose or forehead is often described. In radiation oncology, where there have been dramatic changes concerning automation and technical innovations, the creation of the thermoplastic masks is still a manual process which requires storage space, material, and, most importantly, human resources. While 3D-printing is implemented in many industrial branches for the creation of cost-effective production of individual 3D-objects [1], even in the field of medical applications [18], it is still used only scarcely in radiation oncology. The most common applications are the creation of custom beam range modulation devices [4, 13], devices for dosimetry [7, 22], or brachytherapy applications [5, 14]. With the huge potential of 3D-printing, it may be possible to overcome some of the abovementioned disadvantages of the currently used fixation systems. The production of these new innovative fixation systems in our department has been described before [10]. While the preceding study focused on the creation and position accuracy of this innovative fixation device, the prevailing study is the first to test the new fixation device in a clinical setting as an individual treatment on a small cohort receiving whole brain radiotherapy (WBRT). In terms of translation from bench to bed, this is a crucial step and indispensable concerning a possible regular use in therapy. To the best of our knowledge, our group is the first to have developed an innovative and practicable repositioning system using a 3D-printer, which has been validated in a proband trial and has now been used in a clinical setting for the first time [10].

In the present study, we compare a small cohort of patients undergoing whole brain radiotherapy in the new mask system to patients undergoing radiotherapy in a standard system in the same clinical setting.

## Material and methods

Six patients (all women) with the indication for WBRT received an individual mask system created in the previously described technique [10]. Using those individual masks, a planning CT scan was performed using a Siemens Somatom Sensation Open CT system (Siemens, Erlangen, Germany). Treatment planning was performed using the tomotherapy planning station (Accuray, Sunnyvale, CA, the USA). The fact that only women were treated with the new mask system was by coincidence. All eligible patients were offered the study treatment, and the first to consent was all women.

All patients underwent WBRT with 10–12 fractions on a tomotherapy unit. MV-CT scans for position verification were performed before each fraction and compared to the initial planning CT scan. Position correction was performed in a two-step process. First an automatching using the provided bony match algorithm in the software was performed. After a careful review of the automatching result—if necessary—a manual 4-DOF (degrees of freedom) correction was performed by the technicians according to the in-house standards. The corresponding lateral (*x*), vertical (*y*), longitudinal (*z*), and rotational correction vectors were noted after each fraction. The matching results were printed out and checked by a physician on a regular basis.

To compare correctional vectors, two retrospective control groups were established. To possibly minimize confounders concerning the workflow (i.e., positioning, planning, image registration) and concerning different teams of technicians, we selected patients that were treated at the same machine as the experimental group. The first group consisted of the chronologically last ten patients who underwent WBRT in 10–12 fractions at the same machine using a standard thermoplastic head fixation system.

For better comparability, a second control group was established, consisting of ten patients who underwent radiotherapy of the head and neck area in 12–25 fractions using a shoulder fixating thermoplastic mask system. For that, we also selected the chronologically last patients.

For both control groups, daily MV-CT scans were performed as well. The matching was performed in the same way as in the experimental group, and the vectors were noted and compared to those of the experimental group using the 3D-printed fixation system.

We compared the absolute values of the correctional vectors to avoid possible confounders (i.e., wrong positioning of the headrest, wrong calibration of the machine table) using descriptive statistics. As the head and neck patients mostly received more than 10 fractions, we only took the first 10 fractions into account, as the correction vectors tend to be bigger toward the end of the treatment due to side effects like weight loss or swelling [12].

The process of the mask production has been described before [10]. The mask system consists of two parts: the individual mask itself and a headrest. Based on image data, an individual mask was produced for each patient, while the headrest was standardized. The headrest can be connected to the treatment table using indexing bars which connect to special bores on the bottom side of the headrest. The upper side provides a frame with a latching mechanism to fasten the individual mask.

**Fig. 1:**
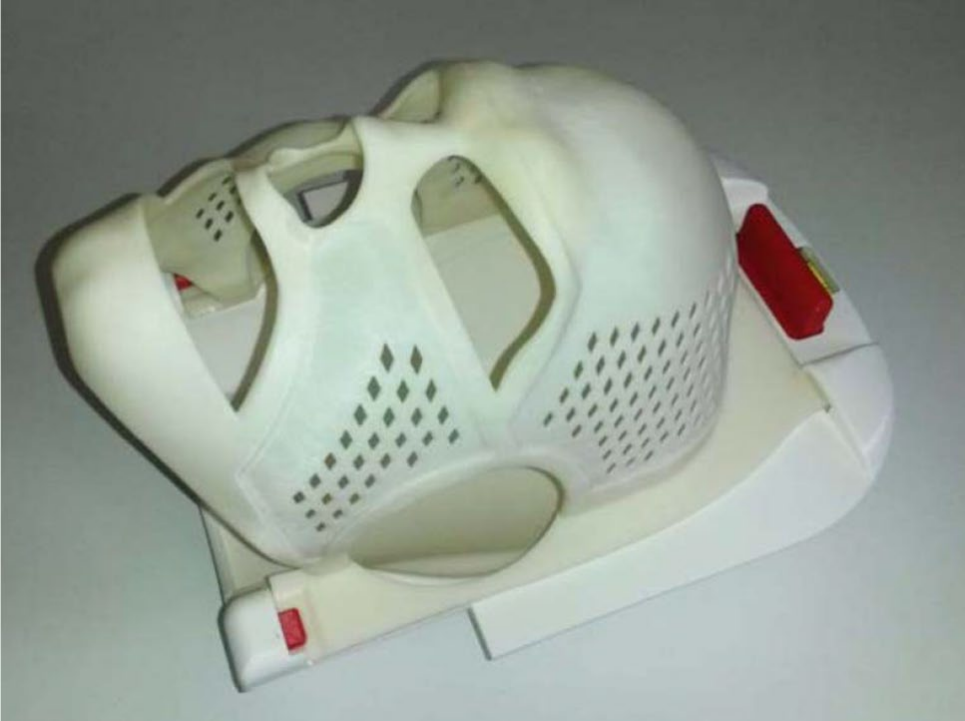
Example for the produced masks

For the mask production, an individual MRI of each patient was performed while using a reusable standardized headrest. Initial imaging was performed using a Magnetom Avanto 1.5 T MRI system (Siemens, Erlangen, Germany) equipped with a 12-element head matrix coil and 4-element neck matrix coil combination. Within about 6 min, a single sagittal image stack in a standard T1-weighted magnetization-prepared rapid gradient echo sequence (MPRAGE) was acquired. Based on the MRI-dataset, an individual 3D-mask was printed using acrylonitrile butadiene styrene (ABS) plastic in fused deposition modeling (FDM) technique, using an in-house developed software and a Stratasys Dimension SST1200es 3D-printer (Stratasys, Eden Prairie, MN, the USA). The masks had a thickness of 1.5 mm and were reinforced by an additional 1 mm around the apertures and the outer mask parts (Fig. 1). The backside of the mask is compatible to the abovementioned headrest.

## Results

In order to show the displacement, the absolute values of the lateral (|Δ*x*|), vertical (|Δ*y*|), longitudinal (|Δ*z*|) correction were calculated. The mean values with standard deviation are listed in Table 1. In addition, the length of the three-dimensional translation vector ($\left| {\overrightarrow {t} } \right| = \sqrt {\Delta x + \Delta y + \Delta z}$) was calculated, and the mean values ($\frac{1}{n}\mathop \sum \nolimits_{i = 1}^{n} v_{i}$ with *n* equal to the number of measurements for each group accordingly and *v* as placeholder for the desired correction value) with standard deviation ($\sqrt {\frac{{\mathop \sum \nolimits_{i = 1}^{n} \left( {v_{i} - \overline{v} } \right)}}{n - 1}}$ with $\overline{v}$ as mean value) for each group are also listed in Table 1. Also, the non-absolute values for the above were calculated and are listed in Table 2. As for the small group size, it is not feasible to make a statistical relevant split between random and systematic error. As an estimation if there was any systematic error, we calculated the non-absolute values. Theoretically with a big enough group size the value this correction converges to would show if there was any shift in the data if not converging to 0. (e.g., if y correction would converge to − 3 we could speak of an existing systematic error). The absolute correction on the other hand is showing the error of our fixation in general. The smaller it is, the better. For our test group, the mean offset was 4.66 mm (std.dev. 1.65 mm), for the WBRT control group we did find 3.61 mm (std.dev. 1.80 mm) and for the head and neck control group 4.29 mm (std.dev. 1.43 mm).

**Table 1:** Average values for absolute lateral (|∆*x*|), vertical (|∆*y*|), longitudinal (|∆*z*|), and rotation correction (roll), as well as the average length for the translational vectors ($\left| {\vec{t}} \right|$)

	|Δ*x*| [mm]	|Δ*y*| [mm]	|Δ*z*| [mm]	Rotation [°]	$\left| {\vec{\user2{t}}} \right|$[mm]
Test group	1.86 ± 1.26	2.91 ± 2.03	2.28 ± 1.32	0.41 ± 0.60	4.66 ± 1.65
Wbrt control group	0.90 ± 0.75	1.90 ± 1.53	2.28 ± 1.97	0.63 ± 0.82	3.61 ± 1.80
Head and neck control group	1.26 ± 0.99	1.58 ± 1.25	3.30 ± 1.76	0.58 ± 0.78	4.29 ± 1.43

**Table 2:** Average values for non-absolute lateral (∆*x*), vertical (*∆y*), longitudinal (∆*z*), and rotation correction (roll)

	Δ*x* [mm]	Δ*y* [mm]	Δ*z* [mm]	Rotation [°]
Test group	0.59 ± 2.18	1.31 ± 3.31	2.10 ± 1.61	0.59 ± 0.72
Wbrt control group	0.18 ± 1.16	0.68 ± 2.35	0.22 ± 3.01	0.18 ± 1.02
Head and neck control group	0.34 ± 1.57	0.26 ± 2.00	2.96 ± 2.28	0.34 ± 0.94

Because the test group only included six patients, the mean values were susceptible for statistical stray. For a better comparability, we also listed the range of the correction values in Table 3. translational systematic errors in that vol

**Table 3:** Minimum and maximum values for lateral (∆*x*), vertical (∆*y*), longitudinal (∆*z*) and rotation correction (roll)

	Min Δ*x* [mm]	Max Δ*x* [mm]	Min Δ*y* [mm]	Max Δ*y* [mm]	Min Δ*z* [mm]	Max Δ*z* [mm]	Min rotation [°]	Max rotation [°]
Test group	− 5	4	− 9	6	− 6	3	− 2	2
Wbrt control group	− 2	3	− 7	5	− 5	9	− 4	3
Head and neck control group	− 3	4	− 6	6	− 4	7	− 3	3

**Fig. 2:**
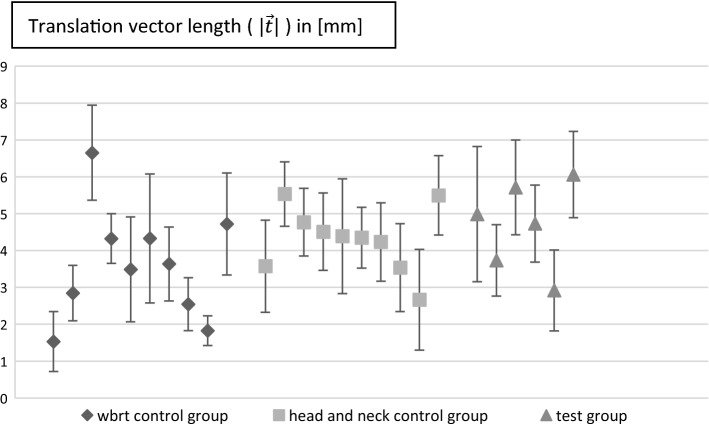
Mean values with standard deviation of each patient for translation vector length ($\left| {\vec{t}} \right|$)

The mean values for the length of the translation vector and the rotation correction of each patient are both plotted in Figs. 1 and 2.

**Fig. 3:**
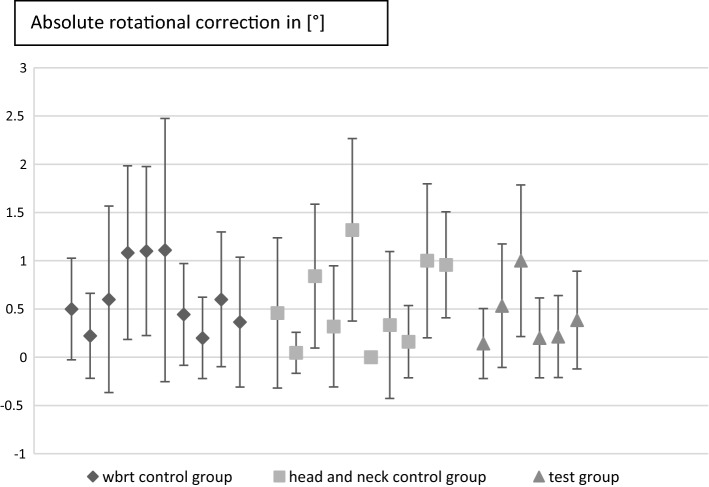
Mean values with standard deviation of each patient for absolute rotational correction ($\left| {\vec{t}} \right|$)

## Discussion

The prevailing study showed the practicability of the mask system in a clinical routine. Furthermore, the positioning was reproducible and within the range of expectancy concerning the positioning uncertainties. Before using the mask system in a clinical setting, the practicability was tested in an experimental setting using volunteers [10]. The translational systematic errors in that volunteer study ranged between − 1.8 and 2.4 mm which is in the range of expectancy of thermoplastic masks and the reason for translating the system in a clinical setting [2]. Those results were confirmed by the prevailing study, which showed an absolute systematic error in the range of 0.59 mm–2.10 mm. It seems that the 3D-printed masks have a reliable accuracy which is rather close to the accuracy of the established mask systems. No additional fitting sessions were necessary between the printing process and the treatment start (Fig. 3).

To the best of our knowledge, this is the first study to use 3D-printed head fixation systems in a clinical setting for WBRT. The current study shows that the 3D-printed immobilization devices have a good interfractional repositioning accuracy. A sufficient and reliable accuracy could be seen in all of the six-treated patients. Considering the deviation vectors of established solutions, those of our experimental system appear to be slightly bigger, with the biggest deviation in the vertical direction. No clinical explanation for this phenomenon could be found and it may be biased due to the small patient number. This will be subject to further investigation. In any case, a high level of cautiousness is mandatory in terms of comparative assessment of positioning accuracy. This high level of cautiousness is also the reason why only six patients were treated with the new mask system. Concerning the small sample size in the experimental group as well as the standard group(s) we did not aim for showing any significant differences. The results should be interpreted as a cautious comparison of the innovative new system to an established system. Further investigations with larger groups are mandatory before using the new system in a clinical routine. Nevertheless, as the results are within the millimetric range, the new mask system proved to be reliable without drastic and possibly dangerous interfractional position changes and the next step of testing the system in a bigger number of patients seems to be reasonable.

In the prevailing study, we were only able to assess interfractional positioning accuracy. With the abovementioned resources of the standard MV-CT scan of the tomotherapy, an evaluation of the intrafractional positioning accuracy was not possible. As the interfractional accuracy as shown to be reliable, it could be extrapolated that intrafractional accuracy is also acceptable. Nevertheless, as no post-treatment or mid-treatment scans have been performed, this hypothesis cannot be proven and will be subject to further investigation. Regarding dosimetric differences, the possible variations should be covered by the normally applied PTV safety margin and should not make any significant difference in big target volumes like a WBRT [21]. Concerning smaller highly conformal target volumes (e.g., stereotactic radiosurgery) further investigations and validations need to be performed before taking the next step from bench to bed.

Because of mask design and further possibilities in individualization (e.g., bigger holes for eyes, mouth, and nose), the new 3D-printed mask systems have potential in increasing patient comfort in comparison to the standard thermoplastic mask systems [15, 16]. In addition to that, the uncomfortable part in the creation process where the wet blank is pulled over the patients’ face is redundant. Especially patients who suffer from claustrophobia could benefit from those features. As the current study was a pilot project that focused on practicability, no standardized assessment concerning patient comfort was carried out. This will be subject to further investigations.

Furthermore, the creation process can be automatized, as the patient does not need to be present in the creation process as with the thermoplastic head masks. After taking the needed information the mask can be printed and will be ready when the patients arrive for their planning CT scan. This could be of benefit in the clinical routine, as the time intervals for the planning CT scan can be shorter that with the creation process of the conventional thermoplastic mask systems.

In terms of the clinical workflow, the headrest system could be applied very easily, the patient positioning on the accelerator couch was comparable to the one using thermoplastic masks with the click-in mechanism between the mask and the headrest. As the handling of the mask system does not differ much from the standard thermoplastic mask system, it was described as practicable by the technicians at the machine. In our daily routine, we did not need to reserve longer time slots for the patients with the new mask system. In terms of the important time-consumption during daily routine, the system seemed to be comparable to the standard thermoplastic system. Furthermore, we did not get any negative feedback from any of the patients treated in the new mask system.

As it was a pilot project, the overall clinical workflow was more complex than the established one using thermoplastic masks. The additional MRI-scan alone needs more personal and financial resources as well as time. We are well aware of those limitations and are currently trying new methods to optimize the workflow by using optical scanners. Like that the patient’s information could be acquired in the outpatient department already, the mask could be automatically produced, and afterward, the CT scan could be acquired all in one appointment. A detailed cost-effectiveness analysis has not been conducted as part of this study but will be investigated in the future. Also, the radiotherapy of smaller target volumes than a WBRT should be evaluated. Therefore, an evaluation with more patients will be necessary. Furthermore, an evaluation of the intrafractional positioning accuracy will need to be performed. Last but not least, 3D printing offers a broad spectrum of possibilities considering individual positioning of otherwise hard to fixate body parts like arms or legs. This will be subject to further research.

## Conclusion

The current study was the first use of a 3D-printer-based mask system in an everyday routine patient treatment. The new system showed a reliable and reproducible interfractional positioning accuracy.

Of course, further investigation needs to be performed before the new system can be used on a regular basis.

## References

[1] Berman B (2012). 3-D printing: the new industrial revolution. Bus Horiz.

[2] Boda-Heggemann J, Walter C, Rahn A, Wertz H, Loeb I, Lohr F, Wenz F (2006). Repositioning accuracy of two different mask systems-3D revisited: comparison using true 3D/3D matching with cone-beam CT. Int J Radiat Oncol Biol Phys.

[3] Cacicedo J, Perez JF, Ortiz De Zarate R, Del Hoyo O, Casquero F, Gomez-Iturriaga A, Lasso A, Boveda E, Bilbao P (2015). A prospective analysis of inter- and intrafractional errors to calculate CTV to PTV margins in head and neck patients. Clin Transl Oncol.

[4] Canters RA, Lips IM, Wendling M, Kusters M, Van Zeeland M, Gerritsen RM, Poortmans P, Verhoef CG (2016). Clinical implementation of 3D printing in the construction of patient specific bolus for electron beam radiotherapy for non-melanoma skin cancer. Radiother Oncol.

[5] Cunha JA, Mellis K, Sethi R, Siauw T, Sudhyadhom A, Garg A, Goldberg K, Hsu IC, Pouliot J (2015). Evaluation of PC-ISO for customized, 3D Printed, gynecologic 192-Ir HDR brachytherapy applicators. J Appl Clin Med Phys.

[6] Engelsman M, Rosenthal SJ, Michaud SL, Adams JA, Schneider RJ, Bradley SG, Flanz JB, Kooy HM (2005). Intra- and interfractional patient motion for a variety of immobilization devices. Med Phys.

[7] Gallas RR, Hunemohr N, Runz A, Niebuhr NI, Jakel O, Greilich S (2015). An anthropomorphic multimodality (CT/MRI) head phantom prototype for end-to-end tests in ion radiotherapy. Z Med Phys.

[8] Gilbeau L, Octave-Prignot M, Loncol T, Renard L, Scalliet P, Gregoire V (2001). Comparison of setup accuracy of three different thermoplastic masks for the treatment of brain and head and neck tumors. Radiother Oncol.

[9] Guckenberger M, Roesch J, Baier K, Sweeney RA, Flentje M (2012). Dosimetric consequences of translational and rotational errors in frame-less image-guided radiosurgery. Radiat Oncol.

[10] Haefner MF, Giesel FL, Mattke M, Rath D, Wade M, Kuypers J, Preuss A, Kauczor HU, Schenk JP, Debus J, Sterzing F, Unterhinninghofen R (2018). 3D-Printed masks as a new approach for immobilization in radiotherapy - a study of positioning accuracy. Oncotarget.

[11] Hong TS, Tome WA, Chappell RJ, Chinnaiyan P, Mehta MP, Harari PM (2005). The impact of daily setup variations on head-and-neck intensity-modulated radiation therapy. Int J Radiat Oncol Biol Phys.

[12] Hoogeman MS, Nuyttens JJ, Levendag PC, Heijmen BJ (2008). Time dependence of intrafraction patient motion assessed by repeat stereoscopic imaging. Int J Radiat Oncol Biol Phys.

[13] Ju SG, Kim MK, Hong CS, Kim JS, Han Y, Choi DH, Shin D, Lee SB (2014). New technique for developing a proton range compensator with use of a 3-dimensional printer. Int J Radiat Oncol Biol Phys.

[14] Lindegaard JC, Madsen ML, Traberg A, Meisner B, Nielsen SK, Tanderup K, Spejlborg H, Fokdal LU, Norrevang O (2016). Individualised 3D printed vaginal template for MRI guided brachytherapy in locally advanced cervical cancer. Radiother Oncol.

[15] Nixon JL, Brown B, Pigott AE, Turner J, Brown E, Bernard A, Wall LR, Ward EC, Porceddu SV (2019). A prospective examination of mask anxiety during radiotherapy for head and neck cancer and patient perceptions of management strategies. J Med Radiat Sci.

[16] Nixon JL, Cartmill B, Turner J, Pigott AE, Brown E, Wall LR, Ward EC, Porceddu SV (2018). Exploring the prevalence and experience of mask anxiety for the person with head and neck cancer undergoing radiotherapy. J Med Radiat Sci.

[17] Partridge M, Powell C, Koopman M, Humbert Vidan L, Newbold K (2012). Technical note: 9-month repositioning accuracy for functional response assessment in head and neck chemoradiotherapy. Br J Radiol.

[18] Rengier F, Mehndiratta A, Von Tengg-Kobligk H, Zechmann CM, Unterhinninghofen R, Kauczor HU, Giesel FL (2010). 3D printing based on imaging data: review of medical applications. Int J Comput Assist Radiol Surg.

[19] Sharp L, Lewin F, Johansson H, Payne D, Gerhardsson A, Rutqvist LE (2005). Randomized trial on two types of thermoplastic masks for patient immobilization during radiation therapy for head-and-neck cancer. Int J Radiat Oncol Biol Phys.

[20] Tryggestad E, Christian M, Ford E, Kut C, Le Y, Sanguineti G, Song DY, Kleinberg L (2011). Inter- and intrafraction patient positioning uncertainties for intracranial radiotherapy: a study of four frameless, thermoplastic mask-based immobilization strategies using daily cone-beam CT. Int J Radiat Oncol Biol Phys.

[21] Van Herk M (2004). Errors and margins in radiotherapy. Semin Radiat Oncol.

[22] Zavan R, Mcgeachy P, Madamesila J, Villarreal-Barajas JE, Khan R (2018). Verification of Acuros XB dose algorithm using 3D printed low-density phantoms for clinical photon beams. J Appl Clin Med Phys.

